# Human amniotic fluid as a source of stem cells

**DOI:** 10.1515/med-2022-0468

**Published:** 2022-04-04

**Authors:** Pawel Walentowicz, Pawel Sadlecki, Malgorzata Walentowicz-Sadlecka, Anna Bajek, Marek Grabiec, Tomasz Drewa

**Affiliations:** Department of Obstetrics and Gynecology, L. Rydygier Collegium Medicum in Bydgoszcz, Nicolaus Copernicus University, Bydgoszcz 85-168, Poland; Department of Tissue Engineering, Nicolaus Copernicus University, Bydgoszcz 85-092, Poland; Department of Regenerative Medicine, Cell and Tissue Bank, Chair of Urology, Nicolaus Copernicus University in Torun, Ludwik Rydygier Medical College in Bydgoszcz, 85-094, Poland; Department of Obstetrics, Gynecology and Oncological Gynecology, Regional Polyclinical Hospital, 87-100 Torun, Poland; 2nd Department of Obstetrics and Gynecology, Centre of Postgraduate Medical Education, 01-809 Warsaw, Poland

## Abstract

Human amniotic fluid collected during amniocentesis contains a heterogeneous population of differentiated and undifferentiated cells. Properties and number of these cells vary depending on the gestational age and the presence of potential fetal pathologies. The aim of this study was to analyze the effects of maternal, fetal, and environmental factors on the success rates of amniotic fluid stem cell cultures, the number of human amniotic fluid stem cells (hAFSC), their growth rates in primary cultures, and the number of cell passages. The study included 355 patients qualified for genetic amniocentesis at the Prenatal Genetic Unit, Department of Obstetrics, Gynecology and Oncologic Gynecology, Nicolaus Copernicus University Medical College in Bydgoszcz in 2011–2017. The mean age of the study participants was 34 ± 6.2 years, and mean gravidity amounted to 2.48 ± 1.4. Amniotic fluid sample volume turned out to be a highly significant (*p* < 0.01) predictor of culture success, and the relationship was particularly evident in women older than 40 years. Another highly significant predictor of culture success was the presence of two cell populations in the sample (*p* < 0.01). The likelihood of culture success correlated significantly (*p* < 0.05) with the season of the year at the time of amniocentesis. The number of cell passages differed significantly depending on the maternal age (*p* < 0.01). The number of passages also showed a highly significant relationship with the season of the year the sample was obtained (*p* < 0.01). Younger maternal age was identified as a determinant of high passage number (≥3), and another highly significant determinant of high passage number was the presence of two cell populations in the amniotic fluid sample (*p* < 0.01). Percentage of successfully established hAFSC cultures and the number of passages depended on amniotic fluid volume, the presence of two cell populations within the sample, and the season of the year. Individual characteristics of the donors, such as age and gravidity, did not exert a significant effect on the number of isolated hAFSCs and the rate of their growth. Patients’ place of residence, fetal karyotype, transportation time, and purity of the samples did not affect the success rates for primary cultures and the number of passages.

## 1 Introduction

The term “stem cells” refers to the cells that are capable of self-renewal, i.e., can undergo unlimited divisions and differentiate into many various cell types [1]. Depending on their differentiation potential, stem cells can be classified as totipotent (i.e., the cells that can differentiate into all the cell types in a body, as well as into extra-embryonic cells), pluripotent (that can differentiate into cells derived from all three germ layers), multipotent (differentiating only into cells characteristic for one specific germ layer), and unipotent (that can turn into only one particular type of cell) [2,3]. Based on their origin, stem cells are classified into embryonic stem cells (ESCs), fetal stem cells (FSCs), adult stem cells (ASCs), and induced pluripotent stem cells (iPSCs) [4]. Despite their relatively lower differentiation potential and limited possibilities of their genetic modification, ASCs represent a potential source of stem cells used for clinical purposes.

Recently, the role of stem cells is extensively examined in the development of different benign and malignant gynecological pathologies. Endometrial stem progenitor cells were proposed as cells that give rise to the origin of benign and malignant endometriotic lesions. Endometrial stem cells demonstrated a high plastic capacity of differentiation by the characterization of several lines of cells with a different expression pattern of cell surface markers, endometrial localization, and clonal efficiency. The endometrial stem cells have demonstrated the ability to migration, adhesion, proliferation, and induction of angiogenesis. It is proposed that physical and biochemical injuries caused by inflammatory cytokines and reactive oxygen species trigger the activation of endometrial stem cells inducing local production of estrogen and tissue injury-repair mechanisms such as cell cycle activation. On that basis, endometrial stem progenitor cells may be involved in the etiopathogenesis of benign and malignant endometrial aberrations such as endometriosis, endometrial hyperplasia, and endometrial cancer [5,6].

Moreover, the possible role of ovarian stem cells in the initiation and progression of ovarian cancer is getting growing attention. Accumulating evidence suggest that stem cells may play pivotal role in recurrence disease. The initial clinical response is primarily due to the therapeutic efficacy of chemotherapy against differentiated cancer cells that constitute the bulk of the tumor, whereas the high rate of recurrence is thought to be due to remaining drug-resistant cells, biologically distinct, identified as cancer stem cells. Current efforts focus on the genetic and cytological definition of cancer stem cells, to guide the development of new diagnostic and therapeutic perspectives [7,8].

Human amniotic fluid collected during amniocentesis contains a heterogeneous population of differentiated and undifferentiated cells. Properties and number of these cells vary depending on gestational age and the presence of potential fetal pathologies. Periodical changes in fetal status may also contribute to changes in the population of amniotic fluid stem cells [9]. In addition, both total cell count and cell viability may differ considerably from sample to sample. For example, the total cell count in the amniotic fluid from the second trimester was shown to vary from 10 to 1,000 cells/µL [10]. The heterogeneity of the cells can also be a consequence of direct contact of the fluid with fetal tissues due to its variable flow between the growing fetus and the amniotic sac. Some cells found in the amniotic fluid were identified as fetal cells derived from the skin or amniotic membrane [10]. Therefore, the amniotic fluid is routinely used for genetic and biochemical testing for various fetal anomalies and sometimes also to establish the sex of a growing fetus [9].

Based on their morphology, cells present in the amniotic fluid can be divided into three types: amniotic fluid-specific cells (AF-type), epithelioid cells (E-type), and fibroblastic-type cells (F-type). The largest group among these types are AF-type cells that account for 60–70% of all cells found in the amniotic fluid. E-type and F-type cells constitute 20–30% and less than 10% of all cells, respectively [11]. While the published terminology used to describe the amniotic fluid cells varies from paper to paper, usually two main populations, amniotic fluid mesenchymal stem cells (AF-MSCs) and amniotic fluid-derived stem cells (AFSCs), are identified [9]. AF-MSCs are postulated to be more abundant among these two types. However, according to the available literature, we conclude that both have the similar properties, and authors describing mesenchymal stem cells isolated from the amniotic fluid use also both terms interchangeably. That is why we decided to focus on the most common term, which is AFSCs, and its population was subjected to all analysis.

MSCs are a population of multipotent stem cells that can differentiate into mesoderm-derived cells, such as adipocytes, chondrocytes, myoblasts, and osteoblasts [12]. However, these cells were also shown to turn into the cells of ectodermal and endodermal lineages [13,14]. MSCs were first identified in the bone marrow, whereby they constitute 0.001–0.01% of all nuclear cells [15]. However, the presence of these cells, with similar properties to those isolated from the bone marrow, was later demonstrated in many adults, fetal, and extra-embryonic tissue [16].

First reports on the presence of a cellular subpopulation with biological properties resembling those of MSCs in the amniotic fluid were published in 2001 [17]. In 2003, Prusa et al. demonstrated that the cells present in the amniotic fluid could express OCT4, a pluripotency marker [10]. In the same year, In’t Anker et al. showed that the amniotic fluid could be a source of multipotent stem cells [18].

The pool of amniotic fluid stem cells includes a population expressing markers of mesenchymal cells, such as CD90, CD105, CD73, and CD166 and a population lacking expression of hematopoietic cell markers, CD45, CD34, and CD14 [19]. These cells constitute 0.9–1.5% of all cells found in the amniotic fluid [19]. The results of a comprehensive analysis of these cells were published in 2007 by De Coppi et al. who isolated a population of CD117+ cells with clonogenic potential [20]. Colony-forming amniotic fluid stem cells are also capable of self-renewal and can maintain the constant telomere length in late passages. Moreover, despite their high proliferation potential, these cells have typical morphological features of MSCs and express pluripotency markers, such as OCT4, NANOG, SSEA3, SSEA4, and c-MYC even up to 25 passages [21]. Moreover, they maintain normal karyotypes in culture and do not form neoplastic tissue *in vivo* [22]. *In vitro* studies demonstrated that these cells could differentiate into the cells derived from all three germ layers, turning into adipocytes, osteocytes, myocytes, as well as into endothelial cells and neurons. Given those findings, MSCs derived from the amniotic fluid were classified into a new type of multipotent cells that combine the characteristics of embryonic and adult stem cells [23]. Amniotic fluid stem cells also have the ability to modulate immune cells. Several research groups demonstrated that these cells showed decreased expression of HLA-DR and proinflammatory molecules and enhanced the activity of anti-inflammatory molecules, e.g., interleukin 10 [24,25]. In addition, they do not express major histocompatibility complex antigens MHC class II and some clusters of differentiation markers, namely, CD40, CD80, and CD86 [21]. These findings suggest that these cells can release immunosuppressive factors in response to the activation of the immune system, which seems vitally important in the context of prevention of the rejection of transplanted amniotic stem cells by the recipient [9].

The aim of this study was to analyze the effects of maternal, fetal, and environmental factors on the success rates of amniotic fluid stem cell cultures, the number of harvested human amniotic fluid stem cells (hAFSC), their growth rates in primary cultures, and the number of cell passages.

## 2 Materials and methods

### 2.1 Patients

This study included 355 patients qualified for genetic amniocentesis at the Prenatal Genetic Unit, Department of Obstetrics, Gynecology and Oncologic Gynecology, Nicolaus Copernicus University Medical College in Bydgoszcz in 2011–2017. Only women with singleton pregnancy were included in our study. Women with multiple pregnancies and after *in vitro* fertilization were excluded from the study. The mean age of the study participants was 34 ± 6.2 years, and mean gravidity amounted to 2.48 ± 1.4. Amniocentesis was carried out at a mean gestational age of 16.4 ± 1.6 weeks. All patients were qualified for amniocentesis after noninvasive testing. Women who were referred to the prenatal genetic unit between 11 and 13 + 6 weeks of gestation underwent the integrated test in which the risk of fetal abnormalities was estimated based on the results of the ultrasound and biochemical tests. The latter included the determination of serum concentration of free beta-hCG and PAPP-A. The results expressed as multiples of the median (MoM) were 2.4 ± 7.4 for beta-hCG and 0.8 ± 1.4 for PAPP-A (the mean values were as follows: 44.18 ± 23.4 IU/l for free beta-hCG and 3.36 ± 2.27 IU/l for PAPP-A). Comprehensive ultrasonographic examination (GE Voluson E8 apparatus with a volumetric probe) was conducted by one of the experienced operators trained according to the FMF and PTG (Polish Society of Gynecologists and Obstetricians) standards. The list of parameters determined during the ultrasonographic examination included crown-rump length (CRL, mm) to confirm gestational age (range 45–84 mm), the presence of nasal bone (NB), nuchal translucency (NT, mm), and fetal heart rate (FHR).

Patients who were qualified for invasive prenatal testing due to medical indications were first consulted by a physician. During the consultation with a specialist in clinical genetics, they were familiarized with detailed indications for amniocentesis and potential complications of this procedure and asked about their preferences. On a scheduled date of the procedure, each patient signed the informed consent form prepared according to the Polish Society of Gynecologists and Obstetricians recommendations. During the consultation, the patients were also informed about the option to culture stem cells isolated from the first collected volume of amniotic fluid, which in line with the current recommendations should be discarded as it cannot be used to determine fetal karyotype. After expressing their consent to participate in the study and signing the informed consent form, 355 enrolled women were qualified for the amniotic fluid analysis.

Amniocentesis was carried out in an outpatient setting under ultrasonographic guidance. After initial ultrasonographic examination, including determination of the fetal heart rate, the position of the fetus, and chorion, an optimal puncture site was selected. Routinely, amniocentesis was carried out without anesthesia and antibiotic prophylaxis. Aseptic preparation included disinfection of the puncture site and ultrasonographic probe, inserting the latter into a sterile cuff, and the use of sterile syringes and ultrasonographic gel. The amniotic fluid at a volume of 15–18 mL was collected with a 20-gauge Brown needle, using a free-hand technique or a guidewire. The needle was inserted in such a way that it did not puncture the chorion, or if not feasible, it punctured the placenta at its thinnest part, avoiding the placental margin and insertion of the umbilical cord. The entire puncture procedure was carried out under ultrasonographic guidance. After the assistant connected the syringe to the needle, the first volume of the amniotic fluid, which is not used routinely to determine the fetal karyotype, was collected and sent to the Department of Tissue Engineering to establish the cell culture. Then, the syringe was replaced by the new one, and another volume of amniotic fluid was collected for the determination of the fetal karyotype. After the amniocentesis was completed and the needle was removed, the fetal heart rate was reassessed. After securing amniotic fluid samples under sterile conditions and labeling the syringes appropriately, they were sent to the laboratory. The patients were instructed to self-control for potential amniotic fluid leakage, bleeding, and uterine contractions. Anti-D prophylaxis was recommended to pregnant women who were Rh negative.

Fetal karyotype was determined at the Department of Clinical Genetics, Nicolaus Copernicus University Medical College in Bydgoszcz (Prof. Jurasz Memorial University Hospital No. 1), based on chromosome banding. The most commonly found abnormalities included trisomy 21, trisomy 18, and trisomy 13.

### 2.2 Methodology of laboratory tests

#### 2.2.1 Isolation and *in vitro* culture of human amniotic fluid mesenchymal stem cells

Collected amniotic fluid, at a 0.1–5 mL volume, was transferred from syringes to tubes under sterile conditions of a laminar chamber. The content of the syringes was inspected macroscopically for contamination, such as residual blood. Then, the tubes were centrifuged at 350×*g* for 10 min, the supernatant was discarded, and the precipitate was suspended in Dulbecco’s Modified Essential Medium (DMEM/Ham’s F12, Sigma, Germany) supplemented with 20% fetal bovine serum (FBS, Sigma, Germany), 10 ng bFGF (Sigma, Germany), and 1% antibiotic solution (penicillin/streptomycin, amphotericin B, Sigma, Germany). The viability of the cells was determined by the trypan blue test. Then, the cells were transferred to 35 mm Petri dishes. The dishes were incubated at 37°C with 5% CO_2_ and constant humidity. The culture medium was replaced every 48 h. The cells were cultured until they reached confluence and then transferred to new Petri dishes.

#### 2.2.2 Passaging of amniotic fluid mesenchymal stem cells

All procedures related to maintenance of *in vitro* cell cultures and cell passaging were carried out under sterile conditions of class II laminar chamber (Jouan, France). The first stage of passaging was the removal of the culture medium; then, the growth surface was rinsed with phosphate-buffered saline (PBS) without calcium and magnesium (Sigma, Germany). Subsequently, the cells were treated with 0.05% trypsin solution (Sigma, Germany) mixed with 0.5 mM EDTA (POCh, Poland) in 1:1 ratio. The cells were incubated at 37°C for 5 min, while the degree of their detachment was controlled under an inverted microscope (Nikon, Japan). The trypsinization was inhibited by adding an equal volume of culture medium; then, the material was transferred to a sterile tube, centrifuged at 350×*g* for 10 min, and the sediment was suspended in 1 mL of the culture medium. The number of viable cells was determined in a hemocytometer based on the result of the trypan blue test. Cell morphology was examined under an inverted phase-contrast microscope (CKX53 Olympus); the cells were classified as epithelioid or fibroblastic type.

#### 2.2.3 Determination of amniotic fluid mesenchymal cell viability

To determine cell viability, an equal volume of 0.4% trypan blue solution (Sigma, Germany) was added to the cell suspension. The mixture was applied onto the Neubauer chamber, and the number of viable cells was determined under an inverted microscope. Viable cells were counted in four squares of the Neubauer chamber, and the cell viability was calculated as follows: *L* = *A*/4 × 2 × 104 × *B*, where *L* is the total number of viable amniotic fluid mesenchymal stem cells and *A* is the number of viable cells in four squares of the chamber.

Assays confirming the mesenchymal nature of isolated stem cells, in accordance with the recommendations of the International Society for Cytotherapy, were performed at the Department of Tissue Engineering, Nicolaus Copernicus University Medical College in Bydgoszcz and published previously [26].

### 2.3 Statistical analysis

Statistical analyses were carried out with PQStat package, version 1.6.4.110. A relationship between maternal age and cell culture success rate was analyzed using a logistic regression model, and by the chi-squared test when the age was converted into a categorical variable. Cell counts at the beginning of culture and following subsequent passages were compared with Mann–Whitney *U* test. A relationship between amniotic sample volume and cell culture success rate was analyzed using a logistic regression model. Effects of grouping variables on cell culture success rate were analyzed with the chi-squared test. Relationships among maternal age, cell count at the beginning of the culture (*P*0), or the number of cell passages were analyzed by the Kruskal–Wallis test, Dunn–Bonferroni post-hoc test, and Jonckheere–Terpstra trend test; also, Spearman’s coefficients of correlation between maternal age and the numbers of cells/passages were calculated. Relationships among various dichotomous grouping variables, cell count at the beginning of the culture (*P*0), and the number of cell passages were analyzed by the Mann–Whitney *U* test. Relationships among the season of the year at the time of amniocentesis, cell count at the beginning of the culture (*P*0), or the number of cell passages were analyzed with the Kruskal–Wallis test and Dunn–Bonferroni post hoc test. Predictors of high passage number (≥3) were identified through the logistic regression analysis, and predictors of cell count at the beginning of the culture (*P*0) were determined through the multiple regression analysis. The results of all tests were considered significant at *p* < 0.05 and highly significant at *p* < 0.01.


**Ethics statement:** The protocol of the study was approved by the Local Bioethics Committee at Ludwik. Rydygier Collegium Medicum in Bydgoszcz (decision no. KB 239/2011), and written informed consent was sought from all participants.

## 3 Results

As shown in Table 1, culture success did not depend on maternal age (*p* > 0.05). No statistically significant differences in culture success rates were observed (*p* > 0.05) on comparative analysis of the results for women from various age groups (Table 2).

**Table 1:** Culture success rates depending on maternal age

	Beta	*p*-Value	Odds ratio	−95% CI	+95% CI
Intercept	1.7194	0.0093	5.5810	1.5286	20.3769
Age	−0.0360	0.0530	0.9646	0.9301	1.0005

**Table 2:** Culture success rates in various age groups

Culture success	Age									
	Up to 25		26–30		31–35		36–40		41 and above	
	*N*	%	*N*	%	*N*	%	*N*	%	*N*	%
No	13	32.5	13	27.66	28	34.15	59	45.38	24	42.86
Yes	27	67.5	34	72.34	54	65.85	71	54.62	32	57.14
Chi-square	6.6415									
Df	4									
*p*-value	0.1561									

The amniotic sample volume turned out to be a highly significant (*p* < 0.01) predictor of the culture success. The larger the sample volume, the more likely successful the culture (Table 3). While the relationship was particularly evident in women older than 40 years, it was not observed in patients younger than 25 years (Tables 4 and 5). No significant relationship between the sample volume and culture success was observed in younger women (*p* > 0.05) (Table 4). Among older women, the sample volume was a significant (*p* < 0.05) determinant of culture success. In this age group, the likelihood of culture success increased proportionally to the sample volume (Table 5).

**Table 3:** Culture success rates depending on sample volume (overall)

	Beta	*p*-value	Odds ratio	−95% CI	+95% CI
Intercept	−0.3471	0.0986	0.7068	0.4682	1.0669
Sample volume (mL)	0.4189	<0.0001	1.5203	1.2574	1.8380

**Table 4:** Culture success rates depending on sample volume in women aged 25 years and younger

	Beta	*p*-Value	Odds ratio	−95% CI	+95% CI
Intercept	0.2642	0.7188	1.3024	0.3091	5.4879
Sample volume (mL)	0.2749	0.4844	1.3164	0.6091	2.8452

**Table 5:** Culture success rates depending on sample volume in women older than 40 years

	Beta	*p*-Value	Odds ratio	−95% CI	+95% CI
Intercept	−0.8663	0.0863	0.4205	0.1563	1.1315
Sample volume (mL)	0.6406	0.0111	1.8976	1.1577	3.1102

Another highly significant predictor of culture success was the presence of two cell populations in the amniotic fluid sample (*p* < 0.01); the cultures prepared from the amniotic fluid containing two populations of cells were more likely to be successful (Table 6). While this relationship was not observed among younger women (*p* > 0.05), it was statistically significant in the group of older patients (*p* < 0.05).

**Table 6:** Culture success rates depending on the presence of two cell populations within the sample

Culture success	Overall			
	Presence of two cell populations			
	No		Yes	
	*N*	%	*N*	%
No	121	52.84	13	10.66
Yes	108	47.16	109	89.34
Chi-square	60.0076			
Df	1			
*p*-Value	<0.0001			

Culture success	Younger (<25 years)			
	Presence of two cell populations			
	No		Yes	
	*N*	%	*N*	%
No	12	40%	1	10
Yes	18	60%	9	90
Chi-square	3.0769			
Df	1			
*p*-Value	0.0794			

Culture success	Older (>40 years)			
	Presence of two cell populations			
	No		Yes	
	*N*	%	*N*	%
No	21	56.76	2	11.11
Yes	16	43.24	16	88.89
Chi-square	10.3695			
Df	1			
*p*-value	0.0013			

The likelihood of culture success correlated significantly (*p* < 0.05) with the season of the year at the time of amniocentesis, with the lowest success rates for the samples collected in summer and the highest for the stem cells harvested in winter. The relationship between culture success and the season of the year was not observed in the group of younger women (*p* > 0.05) but was statistically significant among older patients (*p* < 0.05) in whom the highest success rates were obtained for the stem cells harvested in winter (Table 7).

**Table 7:** Culture success rates depending on the season of the year

Culture success	Overall							
	Season							
	Spring		Summer		Autumn		Winter	
	*N*	%	*N*	%	*N*	%	*N*	%
No	38	40.43	30	50.85	30	42.86	39	29.55
Yes	56	59.57	29	49.15	40	57.14	93	70.45
Chi-square	8.9685							
Df	3							
*p*-Value	0.0297							

Culture success	Younger (<25 years)							
	Season							
	Spring		Summer		Autumn		Winter	
	*N*	%	*N*	%	*N*	%	*N*	%
No	5	41.67	2	25	3	42.86	3	23.08
Yes	7	58.33	6	75	4	57.14	10	76.92
Chi-square	1.5332							
Df	3							
*p*-Value	0.6746							

Culture success	Older (>40 years)							
	Season							
	Spring		Summer		Autumn		Winter	
	*N*	%	*N*	%	*N*	%	*N*	%
No	7	41.18	2	28.57	10	76.92	5	26.32
Yes	10	58.82	5	71.43	3	23.08	14	73.68
Chi-square	8.8860							
Df	3							
*p*-Value	0.0308							

No statistically significant relationship was found between the success rates at the time elapsed since amniocentesis to the establishment of the culture (*p* > 0.05). The success rates for cultures established up to 10 h from harvesting and later did not differ significantly (Table 8).

**Table 8:** Culture success rates depending on the time elapsed since amniotic fluid sampling to the establishment of the culture

Culture success	Overall			
	Time elapsed since the sampling			
	<10 h		>10 h	
	*N*	%	*N*	%
No	108	38.85	29	37.66
Yes	170	61.15	48	62.34
Chi-square	0.0358			
Df	1			
*p*-Value	0.8499			

Culture success	Younger (<25 years)			
	Time elapsed since the sampling			
	<10 h		>10 h	
	*N*	%	*N*	%
No	10	29.41	3	50
Yes	24	70.59	3	50
Chi-square	0.9854			
Df	1			
*p*-Value	0.3209			

Culture success	Older (>40 years)			
	Time elapsed since the sampling			
	<10 h		>10 h	
	*N*	%	*N*	%
No	18	42.86	6	42.86
Yes	24	57.14	8	57.14
Chi-square	0			
Df	1			
*p*-Value	1			

The culture success rate was also not associated with contamination of the sample (*p* > 0.05).

Cell count at the beginning of the culture (*P*0) did not correlate significantly with maternal age, sample volume, the presence of two cell populations in the sample, and the season of the year at the time of amniocentesis.

However, multiple regression analysis demonstrated that *P*0 correlated strongly (*p* < 0.01) with the sample volume (Table 9).

**Table 9:** Predictors of high passage number at the beginning of the culture (*P*0)

	Beta	*p*-value	Standardized beta	Standard error for beta
Intercept	327077	0.4912		
Age	12,842	0.3010	0.0538	0.0519
Sample volume (mL)	−205,548	0.0005	−0.1835	0.0520
Season	46,518	0.4442	0.0397	0.0519
Time since the sampling	−163,851	0.3597	−0.0477	0.0520

**Figure 1:**
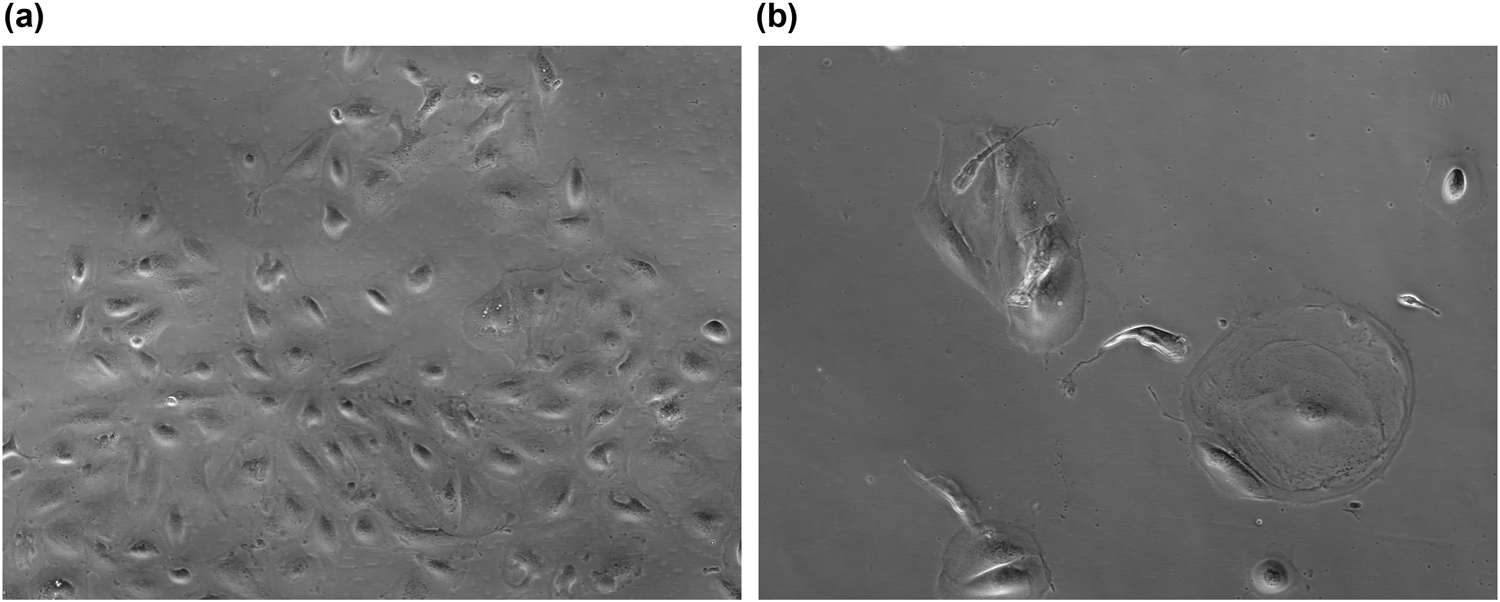
Isolation and *in vitro* culture of hAFSCs. (a) hAFSCs after 2nd passage, (b) hAFSCs after 6th passage.

The number of cell passages differed significantly depending on maternal age; a strong inverse correlation was observed between these two parameters (*p* < 0.01; Table 10). It also differed in morphology and cell density in following passages. In early one, cells were smaller and proliferated very intensively. However, changes in cells’ morphology, their size, and growth were observed in later passages. Human AFSCs were larger and proliferated slower, which can also indicate the undergoing aging process (Figure 1).

**Table 10:** Number of passages in various age groups

	Overall	Up to 25	26–30	31–35	36–40	41 and more
Median	2	1.5	2	2	1	1
Minimum	0	0	0	0	0	0
Maximum	9	7	8	9	9	8
Lower quartile	0	0	0.5	0	0	0
Upper quartile	3	3	2	3	2	2
Kruskal–Wallis test	*H*	7.6830				
	*p*	0.1039				
Jonckheere–Terpstra test	*Z*	2.4620				
	*p*	0.0138				

		Up to 25	26–30	31–35	36–40	41 and more
Dunn–Bonferroni test	Up to 25		1	1	1	1
	26–30	1		1	1	1
	31–35	1	1		0.3933	0.3274
	36–40	1	1	0.3933		1
	41 and more	1	1	0.3274	1	
Spearman correlation	*R*	−0.1361				
	*p*	0.0095				

Moreover, the number of passages was significantly higher when the culture was prepared from a larger volume of the amniotic fluid (*p* < 0.05; Table 11).

**Table 11:** Number of passages depending on sample volume

	Up to 1 mL	More than 1 mL
Median	1	2
Minimum	0	0
Maximum	9	9
Lower quartile	0	0
Upper quartile	2.25	3
*Z*	2.2114	
*p*	0.0270	

A highly significant difference was also found in the number of passages prepared from the amniotic fluid containing one and two populations of cells (*p* < 0.01); in the latter case, the number of passages turned out to be significantly higher (Table 12).

**Table 12:** Number of passages depending on the presence of two cell populations within the sample

	No	Yes
Median	1	2
Minimum	0	0
Maximum	8	9
Lower quartile	0	2
Upper quartile	2	3
*Z*	6.9950	
*p*	<0.0001	

The number of passages also showed a highly significant relationship with the season of the year the sample was obtained (*p* < 0.01). The number of passages for cultures established in winter was significantly higher than for those established in spring and summer (Table 13).

**Table 13:** Number of passages depending on the season of the year

	Overall	Spring	Summer	Autumn	Winter
Median	2	1	1	1	2
Minimum	0	0	0	0	0
Maximum	9	5	5	9	9
Lower quartile	0	0	0	0	0
Upper quartile	3	2	2	2	3
Kruskal–Wallis test	*H*	12.9948			
	*p*	0.0046			

		Spring	Summer	Autumn	Winter
Dunn–Bonferroni test	Spring		1	1	0.0290
	Summer	1		1	0.0191
	Autumn	1	1		0.1372
	Winter	0.0290	0.01914	0.1372	

No statistically significant relationships were observed between the number of passages and sample contamination, fetal karyotype, and indications for amniocentesis (*p* > 0.05).

Younger maternal age was identified as a determinant of high passage number (three and more passages); the older the maternal age, the lower the number of obtained passages. Another highly significant determinant of high passage number was the presence of two cell populations in the amniotic fluid sample (*p* < 0.01; Table 14).

**Table 14:** Predictors of high passage number (at least three passages)

	Beta	*p*-Value	Odds ratio	−95% CI	+95% CI
Intercept	0.2415	0.7329	1.2732	0.3181	5.0968
Age	−0.0478	0.0178	0.9534	0.9164	0.9918
Sample volume (mL)	−0.0093	0.9270	0.9907	0.8120	1.2088
*P*0 (cell count)	0.0000	0.1074	1.0000	1.0000	1.0000
Presence of two cell populations	1.0671	0.0001	2.9068	1.7295	4.8857

## 4 Discussion

The applicability of stem cells for regeneration of tissues and organs is a subject of many currently ongoing studies in the field of tissue engineering and regenerative medicine. In recent years, amniotic fluid has emerged as an alternative source of cells for therapeutic purposes. The uniqueness of this source of stem cells is also associated with the fact that the latter can be used even before birth, in fetal life [27]. Aspiration of the amniotic fluid does not raise controversies of ethical nature as it is a part of a routine amniocentesis procedure. Amniotic fluid stem cells constitute not only a diagnostic instrument but also a potential treatment of many congenital defects [28]. Hence, amniotic fluid stem cells are an interesting research model from a perspective of their applicability in preclinical studies. The volume of amniotic fluid collected for diagnostic purposes is small, approximately 20 mL. If the total volume of amniotic fluid, expressed as an amniotic fluid index (AFI), is normal, amniocentesis does not pose a threat for a growing fetus. Another argument, vitally important from a perspective of the clinical application, is the possibility of storing the sample frozen, even if the pregnancy was terminated by the cesarean section [29].

An interesting problem, albeit rarely addressed in the available literature, is a relationship between the maternal age and the possibility to isolate amniotic fluid stem cells and to culture them *in vitro*. In this study, we did not find a significant difference in culture success rates for amniotic fluid stem cells obtained from pregnant women of different ages. In the study conducted by Romani et al., the culture success rate for amniotic fluid stem cells obtained from a group of 35- to 40-year-old women was 40% [30]. In other studies, which analyzed gestational rather than maternal ages, culture success rates reached up to 93% [31]. We also did not find statistically significant differences in the number of amniotic fluid stem cells obtained from women of various ages. Bielec-Berek et al. and Azouna et al. also did not observe a significant relationship between maternal age and the number and viability of fetal stem cells isolated from another source, i.e., umbilical cord blood [32,33]. Similar findings were also reported by Ballen et al. who did not find an association between maternal age and biological properties of stem cells [34].

Our present study demonstrated that the culture success rate for hAFSCs obtained from women older than 40 years depended on the volume of the amniotic fluid sample, whereas no such relationship was observed for the stem cell cultures from younger patients, younger than 25 years. According to the study by Sessarego et al., the culture success rate for stem cells obtained from 2 mL of amniotic fluid was 91%. However, the authors of that study did not analyze the association between the success rate and the maternal age [35]. Further analyses did not show a significant effect of amniotic fluid volume, less than 1 mL or >1 mL, on the number of isolated cells. In the most previous studies, stem cells were obtained from amniotic fluid volumes greater than 1 mL, and also the results of those experiments suggest that the sample volume does not affect the number of isolated cells [31,36].

In our study, the presence of two cell populations within the sample exerted a significant effect on the success of amniotic fluid stem cell culture, especially if the cells were originated from women older than 40 years. The success rates were higher if the sample contained two cell populations, epithelial and fibroblast-like cells. However, no such relationship was observed in the case of stem cells obtained from younger women younger than 25 years. The presence of two cell populations is a common finding at early stages of *in vitro* culture. After a certain number of passages, the culture contains solely the cells with a fibroblast-like morphology [9], i.e., the proper MSCs [37]. Additional cell population present within the sample might promote proliferation or migration of stem cells, acting in a paracrine manner. However, regardless of the maternal age, we did not observe a significant effect of the additional cell population on the number of isolated amniotic fluid stem cells.

The success rate of hAFSCs cultures depended on the season of the year when amniocentesis was conducted. Irrespective of the maternal age, the highest success rates, more than 70%, were observed for the cultures established in winter. The relationship between the season of the year and culture success rate was statistically significant in older women, older than 40 years, but not in the younger group, younger than 25 years. Moreover, no statistically significant differences were observed in the number of amniotic fluid stem cells isolated in various seasons of the year. While the published data about the isolation of stem cells and their biology are sparse, seasonal and circadian physiological fluctuations are a common phenomenon. Seasonality, e.g., seasonal changes in air temperature, might exert an effect on body weight and reproductive and immune functions; these fluctuations are controlled primarily by the endocrine system. Also, concentrations of hormones in body fluids frequently show circadian or seasonal rhythms. Garde et al. observed seasonal variability in concentrations of total cholesterol, dehydroepiandrosterone sulfate (DHEA-S), prolactin, and free testosterone in healthy women [38]. According to the study by van Anders et al., the highest concentrations of testosterone in women were found in samples collected in autumn and winter [39]. It cannot be excluded that those seasonal fluctuations might also exert an effect on the success rates of amniotic fluid stem cell cultures.

Regardless of the maternal age, sample transportation time, i.e. the time elapsed since the collection of amniotic fluid to the establishment of hAFSC culture, did not exert a significant effect on the culture success rate. Culture success rates exceeded 60% whether the transportation time was below 10 h or above. Whenever the time elapsed since amniocentesis to the isolation of hAFSCs was longer than 10 h, the samples were always stored at 4°C, which probably contributed to the good quality of the amniotic fluid. Pamphilon et al., who analyzed the effect of transportation time on progenitor cells from umbilical cord blood, also concluded that a primary factor determining culture success and influencing the number of isolated cells was storage temperature, rather than the time elapsed since the sampling to the establishment of the culture [40]. Similarly, Matsumoto et al. did not find significant differences in the number of adipose tissue-derived stem cells isolated after various storage times [41]. Published data about optimal storage time, transportation time, and conditions are sparse not only for amniotic fluid stem cells but also for the stem cells from other sources. A comprehensive analysis-based recommendation regarding both storage and transport of the stem cells seems to be of utmost importance, especially in the context of potential clinical applications of this material. Our findings suggest that 4°C is an optimal storage temperature for amniotic fluid, but due to safety concerns, hAFSCs should be isolated immediately after amniocentesis and amniotic fluid collection.

Our study did not reveal a statistically significant relationship between hAFSC culture success rate and purity of the amniotic fluid sample. We observed a tendency to higher success rates of cultures prepared from a noncontaminated amniotic fluid, especially in the case of older women. The number of isolated cells turned out to be significantly higher for contaminated amniotic fluid samples. Contamination of amniotic fluid with the blood affects the initial number of isolated cells. In this study, we used the isolation method based on adherent properties of hAFSCs, which after multiple passages enabled us to select a clone of fibroblastic-type cells and, hence, to minimize the effect of initial sample contamination.

Further analyses did not show an effect of fetal karyotype on hAFSC culture success rates. Without stratification according to maternal age, the success rates for cultures prepared from maternal amniotic fluid from pregnancies with normal and abnormal fetal karyotype were similar. However, the success rates in the group of older women (over 40 years of age) were higher whenever an abnormal karyotype was found in the fetus. Nevertheless, this observation should be interpreted with caution as pregnancies with abnormal karyotype constituted only 11% of the study material. Furthermore, abnormal karyotype exerted no effect on the number of isolated amniotic fluid stem cells. These findings imply that amniotic fluid stem cells with the same karyotype as fetal one can be successfully cultured *in vitro*. This observation seems particularly important from a perspective of research on chromosomal aberrations or mutations accounting for monogenic disorders. According to the study by Rosner et al., mutation-harboring amniotic fluid-derived stem cells can serve as a model for research on many various disease entities [42].

hAFSCs culture success rates for women living in smaller towns, up 50 000, and those from cities over 50 000 were similar. However, it needs to be stressed that a statistically insignificant tendency to higher success rates among younger women living in larger cities was observed. Nevertheless, also this result should be interpreted with caution considering the small size of this group. The lack of statistically significant association between the place of residence and culture success rate should be regarded as a promising finding, especially given published data on the influence of environmental pollution on human health [43]. Stock and Clemens demonstrated that exposure to environmental pollutants might contribute to low birthweight, preterm labor, and respiratory diseases in a growing fetus [44]. While we did not analyze birth-related parameters and postnatal data in this study, the place of residence had no significant effect on the number of isolated cells, their morphology, and the proliferation potential.

We also did not find a significant association between maternal gravidity and culture success rates. Primiparas and multiparas did not differ significantly in terms of hAFSCs culture success rates. Since the amniotic fluid contained also fetal cells, among them exfoliated amniotic cells, one can assume that the sample cellularity, and hence, culture success might depend on the fetal growth and well-being, rather than gravidity [28].

Amniocentesis is used as a prenatal test in clinical practice since the 1950–60s. Initially, the primary indication for this procedure was advanced maternal age defined as more than 35 years [45]. This age limit was specified based on the observed association between older maternal age and increased likelihood of fetal chromosomal aberrations and some diseases, e.g., Down syndrome. In this study, we verified if the stem cell culture success was associated with any of the established indications for amniocentesis. The first analyzed indication was maternal age. Culture success rates in women referred to amniocentesis because of advanced age, and those without this indication amounted to 57% and 66%, respectively, and did not differ significantly. Another analyzed indication was an abnormal result of the integrated test and triple test. In the case of an abnormal result of the integrated test, culture success rates were higher, approximately 68%. However, the success of amniotic fluid stem cell culture did not depend on the result of the triple test. We also did not find statistically significant relationships between culture success rate and another two indications for amniocentesis, evidence of ultrasonographic abnormalities, and fetal hydrops. The last analyzed indication was remarkable obstetrical history. This indication turned out to be a highly significant determinant of culture success rate in the study group. The success rates in women referred to amniocentesis because of remarkable obstetrical history were significantly lower than in other participants of the study. However, this finding should be interpreted with caution as women with the remarkable obstetrical history accounted for only 6% of the study group.

## 5 Conclusion

1. Percentage of successfully established hAFSC cultures and the number of passages depended on amniotic fluid volume, the presence of two cell populations within the sample, and the season of the year.
2. Individual characteristics of the donors, such as age and gravidity, did not exert a significant effect on the number of isolated hAFSCs and the rate of their growth.
3. Patients’ place of residence, fetal karyotype, transportation time, and purity of the samples did not affect the success rates for primary cultures and the number of passages.
